# Plastics in Cyanobacterial Blooms—Genotoxic Effects of Binary Mixtures of Cylindrospermopsin and Bisphenols in HepG2 Cells

**DOI:** 10.3390/toxins12040219

**Published:** 2020-03-31

**Authors:** Klara Hercog, Alja Štern, Sara Maisanaba, Metka Filipič, Bojana Žegura

**Affiliations:** 1 National Institute of Biology, Department of Genetic Toxicology and Cancer Biology, Večna pot 111, 1000 Ljubljana, Slovenia; klara.hercog@nib.si (K.H.); metka.filipic@nib.si (M.F.); 2 Jozef Stefan International Postgraduate School, Jamova 39, 1000 Ljubljana, Slovenia; 3 Area of Toxicology, Department of Molecular Biology and Biochemistry Engineering, University Pablo de Olavide, 41013 Sevilla, Spain; smaiher@upo.es

**Keywords:** cylindrospermopsin, CYN, bisphenols, BPA, BPS, BPF, BPAF, co-exposure, genotoxicity, cytotoxicity

## Abstract

Ever-expanding environmental pollution is causing a rise in cyanobacterial blooms and the accumulation of plastics in water bodies. Consequently, exposure to mixtures of cyanotoxins and plastic-related contaminants such as bisphenols (BPs) is of increasing concern. The present study describes genotoxic effects induced by co-exposure to one of the emerging cyanotoxins—cylindrospermopsin (CYN)—(0.5 µg/mL) and BPs (bisphenol A (BPA), S (BPS), and F (BPF); (10 µg/mL)) in HepG2 cells after 24 and 72 h of exposure. The cytotoxicity was evaluated with an MTS assay and genotoxicity was assessed through the measurement of the induction of DNA double strand breaks (DSB) with the γH2AX assay. The deregulation of selected genes (xenobiotic metabolic enzyme genes, DNA damage, and oxidative response genes) was assessed using qPCR. The results showed a moderate reduction of cell viability and induction of DSBs after 72 h of exposure to the CYN/BPs mixtures and CYN alone. None of the BPs alone reduced cell viability or induced DSBs. No significant difference was observed between CYN and CYN/BPs exposed cells, except with CYN/BPA, where the antagonistic activity of BPA against CYN was indicated. The deregulation of some of the tested genes (*CYP1A1*, *CDKN1A*, *GADD45A*, and *GCLC*) was more pronounced after exposure to the CYN/BPs mixtures compared to single compounds, suggesting additive or synergistic action. The present study confirms the importance of co-exposure studies, as our results show pollutant mixtures to induce effects different from those confirmed for single compounds.

## 1. Introduction

Environmental pollution and accumulation of plastic in the environment are becoming an increasing concern, given the steady rise in plastic production and the release of diverse anthropogenic contaminants into the environment. Organic waste is causing eutrophication of water bodies, which, together with climate change, creates favorable conditions for extensive cyanobacterial proliferation. Cyanobacterial blooms in surface waters are, therefore, globally increasing in extension, frequency, and magnitude [1]. Cyanobacteria produce an impressive plethora of bioactive substances, including a variety of toxins. Among them is the cyanotoxin cylindrospermopsin (CYN), which is considered an emerging health threat worldwide. Compared to other cyanotoxins, humans are more likely to be exposed to CYN, as it is highly water-soluble, very stable and persistent in aquatic environments [2,3], and is predominantly extracellular, especially in older blooms [2,4]. It is produced by a wide variety of species from the genera *Cylindrospermopsis*, *Raphidiopsis*, *Aphanizomenon*, *Chrysosporum*, and *Dolichospermum* (*Anabaena*) [1,5]. The distribution of CYN producing cyanobacteria is expanding globally, also into temperate zones [1,6]. The toxin has been detected in surface fresh and brackish waters in America, Asia, Europe, Oceania, and even in Antarctica, at concentrations up to 200 μg/L (for a review see: [5]). It has even been detected in water used for drinking in the United States of America (USA), China, and Taiwan, at concentrations 0.41 to 36 μg/L [7,8,9]. Furthermore, bioaccumulation of CYN in various aquatic animals and plants has been reported (for a review see: [10]) and the predicted exposure of humans, consuming such organisms, could exceed the provisional tolerable daily intake (TDI) proposed for CYN (0.03 µg/kg body weight) [11].

CYN is an alkaloid with a cyclic guanidine moiety bound to a hydroxymethyluracil group [12]. Its structure alone suggests it could exert a wide range of adverse effects in mammalian cells. In fact, all the main functional groups (uracil, hydroxyl, and guanidine) are crucial for CYN toxicity [13]. CYN is a potent protein synthesis inhibitor [14,15,16] and has been shown to induce oxidative stress [17,18,19]. It has traditionally been classified as a hepatotoxin but has subsequently been shown to target various other organs (for a review see: [20]) and was even reported to have endocrine-disrupting potential [21,22]. The toxin is genotoxic and potentially carcinogenic and needs metabolic activation by cytochrome P450 enzymes to exert genotoxic effects (for a review see: [20,23]). CYN has been shown to induce DNA, and chromosome damage in vitro, suggesting CYN has clastogenic activity [17,24,25,26,27,28]. Its genotoxicity has also been demonstrated in vivo [29,30,31]. Moreover, there are indications that it can also act as a tumor-initiator [29]. However, CYN has not yet been classified for its carcinogenic potential by the International Agency for Research on Cancer (IARC) due to insufficient information on its carcinogenic activity, and the mechanisms potentially involved are still under investigation. 

Concomitantly with the rising emergence of toxic cyanobacterial blooms, the accumulation of plastic in the environment is also increasing. It is estimated that 12,000 million metric tons of plastic waste will be released into the environment by 2050 [32]. Consequently, plastic constituents are becoming ubiquitously present in marine and terrestrial water environments [33], adding to the mixture of present pollutants. A study recently suggested that cyanobacterial blooms could act as a sink for such pollutants [34]. The authors detected three of the plastic-related pollutants bisphenol A (BPA), S (BPS), and F (BPF) in bloom samples in a heavily eutrophic lake in China, at relatively high concentrations of 3954 ng/g dry weight (d.w.), 547 ng/g d.w., and 324 ng/g d.w., respectively.

Among the plastic-related contaminants, BPA is the most common, as it is the most widely-used material in the production of polycarbonate plastics, epoxy resins, and phenolic resins [35]. BPA is an organic synthetic compound belonging to the group of diphenylmethane derivatives, with two hydroxyphenyl groups. The global consumption of this compound in 2016 was estimated to be around 8 million tons, and the global BPA demand is projected to increase to 10.6 million tons by 2022 [36]. However, due to its known endocrine-disrupting activity and its other potential hazardous effects, the use of BPA is being restricted [36], resulting in the gradual replacement of BPA by presumably safer alternatives, its chemical analogues (BPS, BPF, and BPAF). BPS is the most commonly used replacement in various consumer BPA-free products, but BPF and BPAF are used as well in a broad range of industrial applications [37]. 

Due to their massive production, BPs can at present be detected in the environment at alarming concentrations [33,38,39]. They leach off during production, treatment, processing, and hydrolysis of the polymers into the ground water, wastewater, air, and food [40]. BPA tends to elute easily from plastic waste and move rapidly into the aqueous environment, due to its relatively low hydrophobicity (for a review see: [41]). Human exposure is thus unavoidable. The average BPA concentration detected in surface waters is approximately 100 ng/L, but was recorded to be as high as 44,000 ng/L [38]. There are fewer data about the concentrations of BPA analogues, but they are in the range between 1 and 100 ng/L; however, up to 100-fold higher concentrations were detected in the down flow from industrial effluents [33]. Nevertheless, the dietary ingestion of free BPA accounts for its major route of exposure. The daily uptake rate of BPA for humans is estimated to be 50 ng/kg bw/day [42]. It was found to prevail in diverse human tissues and body fluids [43]. Similar data for BPA analogues are scarce. It has to be emphasized that BPA analogue consumption is rising because they are considered safer than BPA. However, the latter assumption is based on insufficient toxicological data to support the risk assessment. Considering the structural similarities and physicochemical properties, BPA analogues are expected to exhibit similar or even stronger endocrine-disrupting and toxic potential as BPA, which is also gradually being confirmed (for a review see: [37]). 

Apart from the well-known endocrine disruption effects of BPA, evidence for its potential genotoxicity is accumulating (for a review see: [37,40]). BPA and its metabolites have been reported to induce DNA strand breaks in vitro [44,45,46] and in vivo [47], chromosomal aberrations in vitro [45,48] and in vivo [45,47,48], and to form DNA adducts in vitro [45,49,50] and in vivo [51]. Similar data for BPA analogues are again scarce. BPF and BPAF were found to induce DNA double-strand breaks in vitro, while BPS was inactive at concentrations up to 20 μg/mL in hepatic cells [46].

Data on the adverse effects of single compound exposure for CYN and BPA are accumulating. However, combined exposure to these pollutants has not been studied thus far. Recently, a review highlighted the importance of BPA co-exposure studies with other chemicals and environmental stressors for the assessment of outcomes that common co-exposures can exert on human health [52]. Considering their simultaneous presence in the environment, exposure to mixtures of different pollutants is the only realistic exposure scenario. Humans can thus be exposed to cyanotoxin/BPs mixtures following recreational activities and/or through the consumption of contaminated water and food. Co-exposure to various pollutants can induce effects that differ from those observed for single compounds due to unknown interactions that can occur between the compounds. The aim of this study was, therefore, to evaluate the cytotoxic and genotoxic potential of mixtures of CYN, BPA, and its commonly used analogues BPS, BPF, and BPAF (Table 1). The co-exposure was studied in the metabolically competent human hepatocellular carcinoma cell line HepG2. This cell line is considered one of the in vitro models of choice when studying the genotoxic effects of progenotoxic agents and is also recommended by Organization for Economic Co-operation and Development (OECD) standards (e.g., 487) [53], as it retained several phase I and II metabolic enzymes, involved in the metabolism of xenobiotics [54]. The genotoxic effects of the mixtures were determined by the detection of H2AX histone phosphorylation, which reflects an early reaction to a genotoxic insult resulting in the formation of DNA double-strand breaks (DNA DSBs). The cellular response to the exposure to these mixtures was further studied by analysis of the transcriptional response—deregulation of selected genes (genes involved in the metabolism of xenobiotics, immediate-early response, and DNA damage response), using qPCR.

## 2. Results and Discussion

### 2.1. The Influence of CYN, BPs, and Their Combinations on Cell Viability

The influence of single compounds and CYN/BPs combinations on HepG2 cell viability was evaluated with the tetrazolium-based MTS assay. The concentrations of CYN (0.5 µg/mL) and BPs (10 µg/mL) were chosen based on previous findings on the genotoxic effects of CYN [25,27,28] and BPs [46] in HepG2 cells, also considering reported environmental concentrations and the significant daily uptake rate through dietary ingestion in the case of BPA. Although these concentrations are still higher than those reported in the environment and are not expected to be directly relevant for human exposure, they serve the aim of this study, which was to identify the possible DNA damaging effects (induction of DNA DSBs) and potential mechanisms of action of non-cytotoxic concentrations of CYN/BPs binary mixtures. It is thought that chemicals, directly inducing DNA damage, have no safe exposure threshold dose, only that the probability of potentially harmful mutations, resulting from the induced DNA damage (e.g., DNA DSBs), decreases with lower concentrations [55].

**Figure 1:**
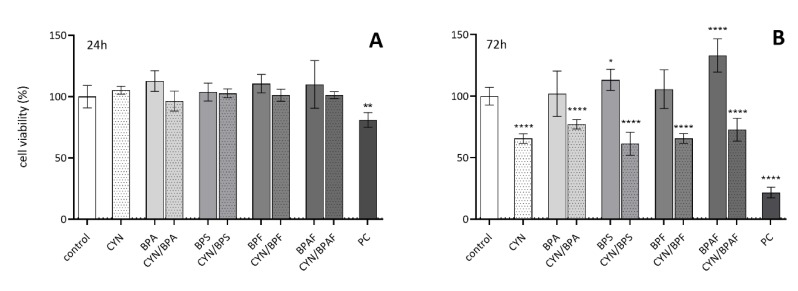
The effects of single compounds and binary mixtures of CYN (0.5 µg/mL) and BPs (BPA, BPS, BPF, BPAF; 10 µg/mL) on the viability of HepG2 cells after 24 h (**A**) and 72 h (**B**) exposure, expressed in percentage of the solvent control (0.05% methanol and 0.04% DMSO = dotted line at value 1.0). PC is the positive control—etoposide ET (30 μg/mL). The lower dotted line in figure B denotes the average viability reduction by CYN. The asterisks (*) denote statistically significant difference between solvent control and treated cells (* *p* ≤ 0.05; ** *p* ≤ 0.01; **** *p* ≤ 0.0001).

After 24 h of exposure, neither single compounds nor the combinations induced any measurable effect on HepG2 cell viability (Figure 1A). At the same time, the positive control (etoposide—ET) reduced cell viability for roughly 16% on average. After longer exposure (72 h), CYN (0.5 µg/mL) and all of its combinations with BPs (10 μg/mL) significantly reduced cell viability by 23% to 38%; however, there was no significant difference between CYN treated cells and cells treated with any of the CYN/BPs combinations (Figure 1B). None of the BPs (10 μg/mL) alone induced a measurable reduction in cell viability. BPS and BPAF even had a slight proliferating or more likely cell metabolic activity enhancing effect, which can commonly be observed after exposure to various toxic agents in cell lines as the consequence of increased mitochondrial activity in cell-cycle arrested cells [56]. Our results are in line with previous findings, showing a reduction in cell viability only after exposure to higher concentrations (15 µg/mL) of BPs (BPA, BPS, BPF, and BPAF) in HepG2 cells [46]. Low cytotoxicity was, for these BPs, also reported in other in vitro model systems (human breast adenocarcinoma cells (MCF-7), human 189 cervical epithelial cancer cells (HeLa), mouse fibroblasts (3T3-L1), and rat glioma cells (C6)), with the calculated half-maximal inhibitory concentrations (IC_50_) generally being in the range of 20–75 µg/mL [57]. The exception was BPAF, which was slightly more toxic (IC_50_: 4–20 µg/mL) in some of the cell models. Thus, we can conclude that the decrease in cell viability observed in cells exposed to the CYN/BPs combinations was the consequence of CYN activity. CYN was previously shown to decrease cell viability and cell proliferation in HepG2 cells after prolonged exposure (96 h) at concentrations of up to 0.5 µg/mL, which was found to be due to the induction of cell cycle arrest rather cell death [28]. Therefore, the observed effects of CYN and the CYN/BPs combinations in this study were probably predominantly the consequence of cell cycle arrest induced by CYN. Compared to the control, CYN at 0.5 µg/mL and CYN/BPs combinations did not reduce cell viability for more than 40%, which is considered the limit value for genotoxicity assessment; thus, these concentrations were used for further studies of their genotoxic effects.

### 2.2. Induction of DNA Double-Strand Breaks by CYN, BPs, and Their Combinations

DNA DSB induction in HepG2 cells after exposure to CYN, BPs, and the CYN/BPs combinations was measured indirectly through the measurement of γ-H2AX formation, using flow cytometry. γ-H2AX is the phosphorylated form of the histone H2AX, which becomes phosphorylated on serine residue 139 in response to DNA DSB induction and forms nuclear foci adjacent to the sites of the DSBs [58]. Because the phosphorylation of H2AX is rapid, abundant, and correlates well with the number of DSBs [59], it is a very sensitive marker for DNA DSB induction (for a review see: [60]).

**Figure 2:**
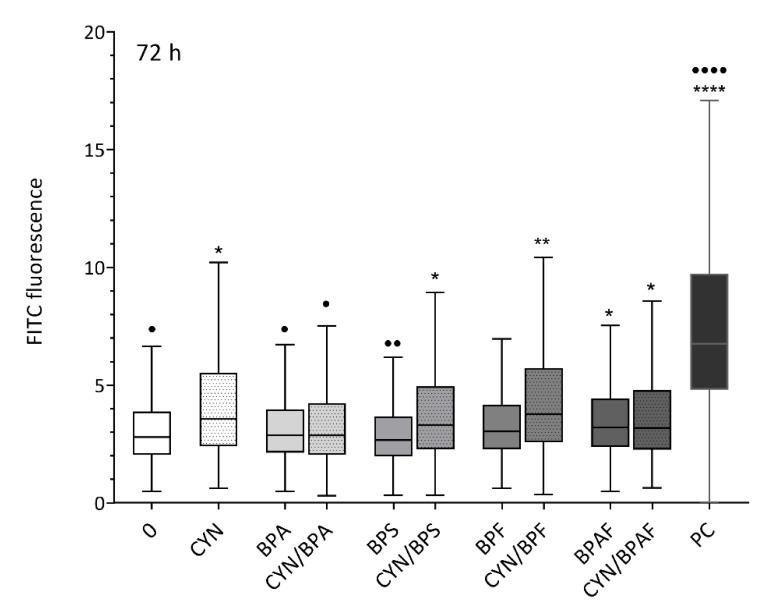
The effect of single compounds and binary mixtures of CYN (0.5 µg/mL) and BPs (BPA, BPS, BPF, BPAF; 10 µg/mL) on the induction of γ-H2AX formation (a sensitive marker for DNA double-strand breaks (DSBs)) in HepG2 cells after 72 h of exposure, using flow cytometry. The data are presented as quantile box plots. The edges represent the 25th and 75th percentiles, the solid line through the box is the median, and the error bars represent 95% confidence intervals. The solvent control (0 = 0.05% methanol and 0.04% DMSO) and a positive control (Etoposide ET, 1 μg/mL) were included in the experiment. The asterisks (*) denote statistically significant difference between solvent control and treated cells (* *p* ≤ 0.05; ** *p* ≤ 0.01; **** *p* ≤ 0.0001). The dots (•) denote a statistically significant difference between CYN alone and CYN/BPs treated cells (• *p* ≤ 0.05; •• *p* ≤ 0.01; •••• *p* ≤ 0.0001).

No increase in γH2X formation was detected after 24 h exposure to CYN, BPs, or CYN/BPs combinations (data not shown). After 72 h of exposure, a significant increase in DSB formation was observed in cells exposed to CYN and its combinations with BPS, BPF, and BPAF (Figure 2). CYN-induced DNA DSB formation in HepG2 cells has also been reported in a previous study [28]. No induction of γH2X was detected in cells exposed to the tested BPs alone except for BPAF, which slightly increased DSB formation. Additionally, in our previous study [46], no induction of DSBs in HepG2 cells at comparable exposure conditions by BPs was observed. BPF and BPAF were found to increase the formation of γH2X at higher concentrations (≥10 μg/mL) or at an earlier time point (24 h). Similarly, Audebert et al. [61] reported that BPA does not induce γ-H2AX in HepG2 cells, human renal cell adenocarcinoma cells (ACHN), and human epithelial colorectal adenocarcinoma cells (LS174T), while BPF (10–50 μM) induced DNA DSBs in HepG2 cells, but not in ACHN and LS174T cells. Comparing the effects of CYN alone and its combination with BPS, BPF, or BPAF, on DSB formation, no significant difference could be detected, indicating that the formed DSBs were the consequence of CYN activity. Interestingly, CYN in combination with BPA induced slightly but statistically significantly less DNA DSB compared to the induction by CYN alone, indicating an antagonistic effect of BPA.

CYN is considered to be a pro-genotoxin [17,18,24,25,62], predominantly activated by CYP450 enzymes [62,63]. BPA is also known to be metabolized by phase I and II xenobiotic-metabolizing enzymes [64,65]. Furthermore, BPA was reported to suppress or inhibit certain human hepatic cytochrome P450s activities [66,67,68]. Thus, the observed antagonistic effects of BPA in the CYN/BPA combination may be due to BPA-mediated suppression of the metabolic activation of CYN.

### 2.3. Gene Deregulation in Response to CYN/BPs Exposure

To get an insight into the response of HepG2 cells to the exposure to CYN in combination with BPs at the molecular level, expression of the metabolic enzyme gene *CYP1A1* (involved in the metabolism of CYN and BPs), and the expression of selected genes involved in DNA damage (*TP53*, *MDM2*, *CDKN1A*, *GADD45A*) and oxidative stress response (*GCLC*, *GPX1*, *GSR*, *SOD1A*, and *CAT*) was analyzed after 24 h of exposure by quantitative real-time PCR.

### 2.4. Xenobiotic Metabolism—CYP1A1

**Figure 3:**
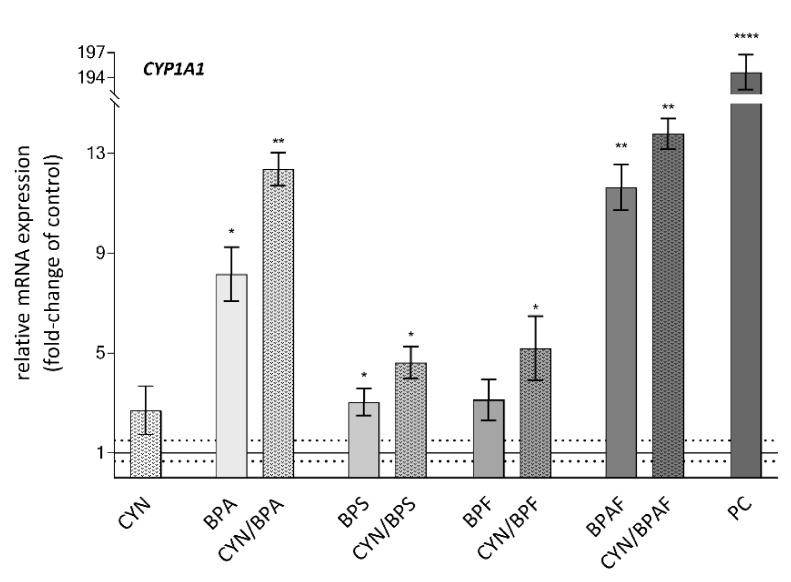
The effect of CYN (0.5 µg/mL), BPs (BPA, BPS, BPF, BPAF; 10 µg/mL) and their binary mixtures on the expression of the *CYP1A1* gene, involved in xenobiotic metabolism, in HepG2 cells after 24 h of exposure. The deregulations are expressed in the fold-change of expression of the gene in the solvent control group (0.05% methanol and 0.04% DMSO = solid line at value 1.0). PC is the positive control—Benzo[a]pyrene BaP (30 μM). The dotted line denotes biologically significant differences in gene expression (1.5-fold change). The asterisks (*) denote a statistically significant difference between solvent control and treated cells (* *p* ≤ 0.05; ** *p* = 0.01; **** *p* = 0.0001).

All of the tested compounds either alone or in combination up-regulated *CYP1A1* expression (Figure 3). The *CYP1A1* gene encodes a member of the family of cytochrome P450 enzymes that are involved in phase I of xenobiotic and drug metabolism. CYN alone up-regulated *CYP1A1* expression by 2.7-fold on average, while the BPs induced an even higher up-regulation. The highest up-regulation was observed in BPAF exposed cells (11.6-fold up-regulation). The exposure to the CYN/BPs combinations exerted stronger response than single compounds and induced *CYP1A1* up-regulation in an additive manner. In the CYN/BPA treated cells, even synergistic action was indicated as *CYP1A1* up-regulation induced by CYN/BPA (12.4-fold) was higher than the sum up-regulation induced by the single compounds (CYN 2.7-fold and BPA 5.3-fold).

Although the main enzymes involved in BPA detoxification are uridine 5′-diphospho-glucuronosyltransferases (UGT) and sulfotransferases (SULT) (for a review see: [66]), they also undergo CYP450-mediated oxidative transformations, as does CYN. The involvement of CYP450 enzymes in the toxic and genotoxic activation of CYN has been demonstrated using different broad-spectrum CYP450 inhibitors, which showed protective effects against toxicity [14,63,69] and genotoxicity [24,62] of CYN. Besides, CYN genotoxic effects were observed only in metabolically competent test systems (for a review see: [20,23]). Furthermore, BPA and BPF have been reported to be oxidized to reactive intermediates as well [70,71], which have been reported to form DNA adducts [51,72,73]. In line with our results, CYN was previously shown to up-regulate *CYP1A1*, and other CYP isoforms (*CYP1B1* and *CYP1A2*) in HepG2 cells [18,25,26,27] and human peripheral blood lymphocytes (HPBLs) [26]. Recently, the up-regulation of the expression of *CYP1A1* was reported following the exposure to BPs (BPA, BPS, BPF, and BPAF) in HepG2 cells [46] and the up-regulation of CYP1A1 on the protein level following exposure to BPA in human placental JEG-3 choriocarcinoma cells was reported [74].

As our results show a more pronounced up-regulation of *CYP1A1* by all tested CYN/BPs combinations compared to the up-regulation of this gene by single compounds, this indicates an even stronger induction of the xenobiotic metabolism. [75,76,77] Meaning that also in addition, CYN and the BPs could potentially increase each other’s genotoxic potency through the increased induction of *CYP* enzymes. However, as the results from the γH2AX assay demonstrate otherwise, showing no further increase in DNA DSB formation in the CYN/BPs exposed; on the contrary, it even reduced in the case of CYN/BPA. Thus, BPs may impair CYN activation through the inhibition of CYP activity.

### 2.5. DNA Damage Response Genes

**Figure 4:**
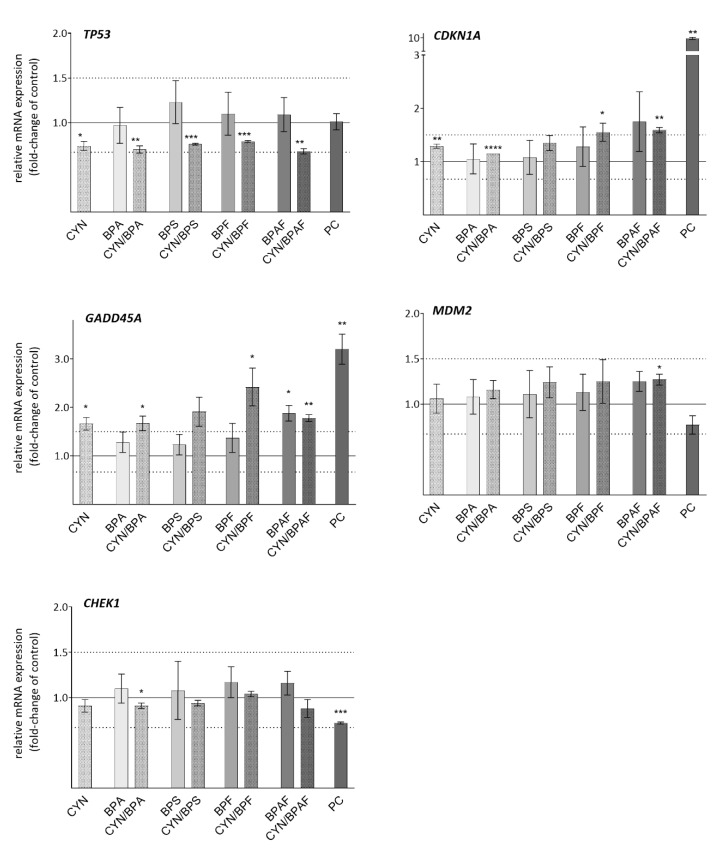
The effect of CYN (0.5 µg/mL), BPs (BPA, BPS, BPF, BPAF; 10 µg/mL), and their binary mixtures on the expression of DNA damage response genes (*TP53*, *MDM2*, *CDKN1A*, *GADD45A*) in HepG2 cells after 24 h of exposure. The deregulations are expressed in fold-change of expression of the gene in the solvent control group (0.05% methanol and 0.04% DMSO = solid line at value 1.0). PC is the positive control—Benzo[a]pyrene BaP (30 μM). The lower and upper dotted lines denote biologically significant differences in gene expression (1.5-fold change). The asterisks (*) denote a statistically significant difference between solvent control and treated cells (* *p* = 0.05; ** *p* = 0.01; *** *p* = 0.001).

Deregulations of crucial DNA damage response genes (*TP53*, *MDM2*, *GADD45A*, *CDKN1A*), which were analyzed in the present study (Figure 4) are considered as molecular markers of genotoxic and carcinogenic stress [78,79,80,81,82]. The protein P53 plays a central role in the major DNA damage response pathways: the regulation of DNA damage repair, cell cycle progression, senescence, differentiation, and apoptosis [83]. In response to DNA damage, P53 protein is predominantly activated through its phosphorylation by DNA damage responsive kinases and, to a lesser extent, through the up-regulation of gene expression [84]. The expression of the tumor-suppressor gene, *TP53*, was not significantly altered by BPs exposure, whereas it was slightly (<1.5-fold) down-regulated by CYN and the CYN/BPs combinations. Previous studies on the expression of the *TP53* gene reported that CYN [18,25,27] and BPs [46] did not influence *TP53* expression in HepG2 cells. Our results indicate that the combined exposure to CYN/BPs does not affect the expression of this gene differently than exposure to CYN as a single compound.

The main P53 down-stream regulated genes are *CDKN1A* and *GADD45A*. The gene *CDKN1A* encodes the cyclin-dependent kinase inhibitor P21^WAF1/CIP1^, an essential regulator of cell cycle progression, and mediator of the P53-dependant G1 and G2 phase arrests (for a review see: [85]). *CDKN1A* was more than 1.5-fold up-regulated only after exposure to BPAF (1.75-fold) alone and the CYN/BPAF (1.59-fold) and CYN/BPF (1.55-fold) combinations. The up-regulation of *CDKN1A* by CYN alone in the present study was only 1.3-fold, which is lower than found previously [18,25].

The protein encoded by *GADD45A* (Growth arrest and DNA damage-inducible protein alpha) is implicated in the control of cell cycle G2-M transition, induction of cell death/survival, DNA repair process, chromatin assembly, and genome stability [86]. In contrast to *CDKN1A*, the expression of the gene *GADD45A* was up-regulated more than 1.5-fold in all CYN exposed groups (CYN alone 1.66-fold; CYN/BPs combinations 1.78–2.42-fold). The BPs alone did not influence *GADD45A* expression, with the exception of BPAF, which up-regulated its expression by approximately 1.9-fold. Among the tested BPA analogues, BPAF was previously found to be the only one that induced up-regulation of the expression of *CDKN1A1* and *GADD45A* and was also the most potent inducer of DNA DSBs in HepG2 cells [46]. Nevertheless, no additive or synergistic effect of BPAF in combination with CYN on the *CDKN1A1* or *GADD45A* expression was indicated. The highest up-regulation of *GADD45A* was observed in cells exposed to the CYN/BPF mixture (2.42-fold), much higher than BPF (1.37-fold) or CYN alone, which suggests synergistic action of the compounds in the mixture.

The MDM2 and CHEK1 genes were the most un-responsive to the exposure to the tested compounds. The expression of the gene encoding the MDM2 protein, a negative regulator of P53 [87], was stable in all the tested cell populations, regardless of the exposure to CYN, BPs, or their binary mixtures. The same was observed with the CHEK1 gene, the protein product of which is a checkpoint kinase that is activated in response to DNA damage and can thereafter modulate the activity of a number of proteins, including P53, providing a link between DNA damage and P53 checkpoint activity [88]. The results observed in single compound exposure groups are in agreement with previous reports, showing no deregulation of these genes by CYN or BPs in HepG2 cells [25,27,46], and the tested binary mixtures did not induce any different deregulation patterns from single compounds.

### 2.6. Oxidative Stress Response Genes

The influence of single compounds and CYN/BPs binary mixtures on oxidative stress induction in HepG2 cells was evaluated by analyzing the deregulation of selected oxidative stress response genes. One of the possible mechanisms of CYN genotoxicity is postulated to be the formation of reactive oxygen species (ROS) that can induce DNA damage. However, the published data are not consistent. While there are studies reporting increased ROS formation by CYN in various test systems, oxidative damage was observed rarely (for a review see: [20,23]).

There is growing evidence for ROS induction by BPA that may contribute to its toxicity and carcinogenic potential (for a review see: [89]). Huc et al. [90] reported mitochondria-dependent ROS generation, cytosolic oxidative stress, and lipid peroxidation in HepG2 cells following exposure to low doses (10^−6^–100 µM) of BPA. Moreover, BPA and its analogues (BPS, BPF, and BPAF) have been reported to induce oxidative stress and oxidative damage in human peripheral mononuclear cells and human red blood cells [91,92].

The reported increase in ROS formation following exposure to CYN and BPs could be, at least in part, the consequence of their metabolic activation. It is known that reactions catalyzed by CYP450 enzymes, which are involved in CYN and BPs metabolism, are a significant source of ROS formation (for a review see: [93]). Given that our results show increased up-regulation of *CYP1A1* by CYN/BPs binary mixtures, a higher increase in ROS formation and enhanced oxidative stress response could be expected in cells exposed to the mixtures compared to single compound exposure. The cellular response to an increase in intracellular ROS formation is the induction of enzymatic and non-enzymatic defensive mechanisms. The enzymes superoxide dismutase (SOD), catalase (CAT), and glutathione peroxidase (GPx) play critical roles in maintaining intracellular redox homeostasis by scavenging and catalytic removal of the generated ROS [94]. The most abundant intracellular antioxidant, central to the non-enzymatic oxidative stress defense, is glutathione (GSH). During oxidative stress, reduced GSH is oxidized to glutathione disulfide (GSSG); therefore, the ratio of GSH to GSSG reflects cellular oxidative stress [95]. Two of the major enzymes involved in the regulation of the intracellular GSH content are glutathione reductase (GSR), which catalyzes the reduction of GSSG to GSH and γ-glutamylcysteine synthetase (GCL), which is recognized as the rate-limiting enzyme in GSH de-novo biosynthesis [96]. Thus, the deregulation of the major antioxidant enzymes and enzymes involved in the GSH detoxification and antioxidant pathways can be considered as a marker for oxidative stress.

**Figure 5:**
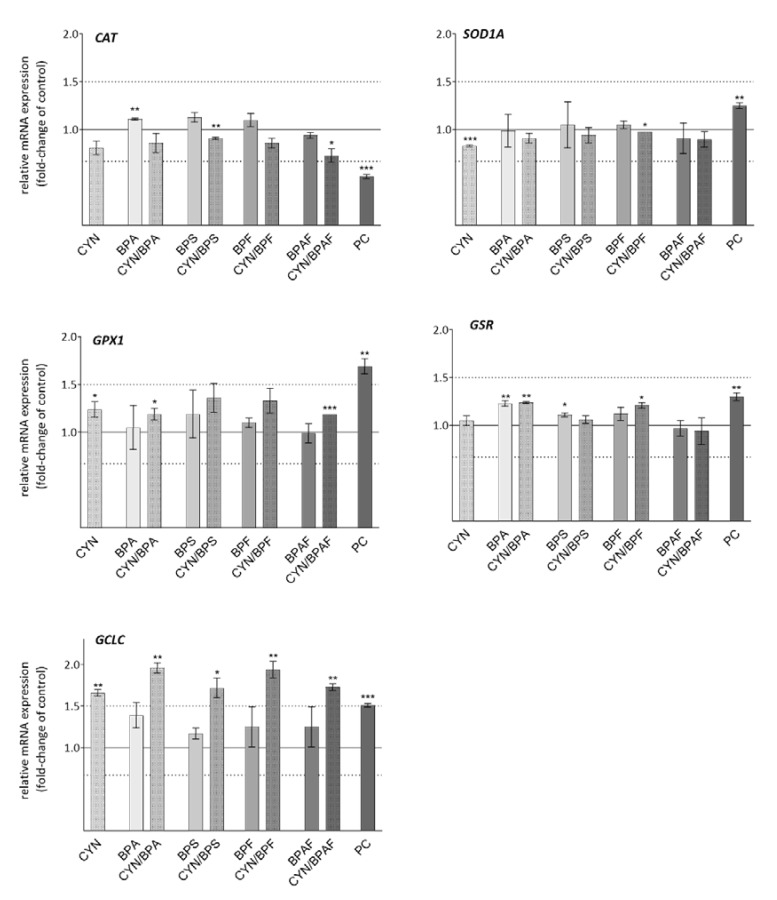
The effect of CYN (0.5 µg/mL), BPs (BPA, BPS, BPF, BPAF; 10 µg/mL), and their binary mixtures on the expression of oxidative stress response genes (*GCLC*, *GPX1*, *GSR*, *SOD1A*, *CAT*) in HepG2 cells after 24 h of exposure. The deregulations are expressed in fold-change of expression of the gene in the solvent control group (0.05% methanol and 0.04% DMSO = solid line at value 1.0). PC is the positive control—Benzo[a]pyrene BaP (30 μM). The lower and upper dotted lines denote biologically significant differences in gene expression (1.5-fold change). The asterisks (*) denote a statistically significant difference between solvent control and treated cells (* *p* = 0.05; ** *p* = 0.01; *** *p* = 0.001).

In the present study, among the tested oxidative response genes (Figure 5), the *GCLC* gene, which encodes the catalytic subunit of GCL, was the most affected by the exposure to CYN and all CYN/BPs mixtures. The gene was significantly up-regulated by CYN (1.66-fold) alone but not by the single BPs (<1.39-fold). The co-exposure to CYN/BPA (1.96-fold), CYN/BPS (1.72-fold), CYN/BPF (1.94-fold), and CYN/BPAF (1.73-fold) up-regulated *GCLC* gene expression to a higher extend then exposure to CYN alone, suggesting additive effects of the compounds in the binary mixtures. *GCLC* gene up-regulation indicates a possible cell response to GSH depletion, increasing its biosynthesis. Decreased GSH content in the cells can result from either increased oxidation, increased efflux, the formation of GSH-conjugates, or decreased synthesis (for a review see: [97]). CYN [62,69] and BPA (for a review see: [89]) have both been shown to reduce intracellular GSH. However, in the case of CYN, the GSH depletion might be the consequence of GSH synthesis inhibition, as has been shown in primary rat and mouse hepatocytes [62,69]. Thus, our results indicate that exposure to the CYN/BPs binary mixtures might reduce intracellular GSH levels in an additive manner.

The other studied oxidative stress-responsive genes were generally not significantly deregulated by the tested compounds and/or the deregulation was less than 1.5-fold (Figure 5). *SOD1* was slightly down-regulated by CYN, while BPs and the combinations did not influence the expression of this gene. There was an indication of slight down-regulation of *CAT* and slight up-regulation of *GPX1* by CYN and all CYN/BPs combinations. Our results suggest that single or combined exposure to CYN and BPs after 24 h in HepG2, at the tested conditions, did not cause major oxidative stress. Except in the case of the gene *GCLC*, there was no indication for additive, synergistic, or antagonistic interactions of the compounds in the binary mixtures that would affect oxidative response gene expression. In line with previous findings [46] in the HepG2 cell line, BPA and its analogues (BPS, BPF, and BPAF) as single compounds had no significant influence on the expression of the selected oxidative stress response genes. On the other hand, the activities of the enzymes SOD, CAT, and GPX were reported to be increased in human erythrocytes following exposure to BPA, BPF, and BPAF, but not BPS [92]. However, changes were observed at 10-fold higher concentrations than used in the present study. Therefore, the slight deregulations of *CAT* and *GPX1* in the cells exposed to the CYN/BPs combinations seem to be the consequence of CYN activity and reflect previously confirmed findings, showing CYN to induce only minor oxidative stress in HepG2 cells at the tested time point and concentration range used [18,19]. In addition, CYN induced a significant increase in the expression of genes *GCLC*, *GSR*, *GPX1*, and *SOD1* after 24 h of exposure in HPBLs. In contrast, the expression of *CAT* was not changed [26], indicating that lymphocytes might be more sensitive to CYN in terms of oxidative stress than hepatocytes.

## 3. Conclusions

In the present study, the cyto/genotoxic effects after co-exposure to emerging aquatic contaminants, the cyanobacterial toxin CYN in combination with BPA, and its analogues BPS, BPF, and BPAF were studied in the metabolically competent HepG2 cell line, for the first time. Exposure to CYN and the CYN/BPs binary mixtures significantly reduced the viability of HepG2 cells and increased the formation of DNA DSBs. The BPs alone did not decrease HepG2 cell viability and had no effect on DNA DSB induction. Generally, no significant differences were observed between cells treated with CYN alone and the CYN/BPs combinations, suggesting that the observed effects were the consequence of CYN activity. The only exception was the CYN/BPA combination, where significantly lower DNA DSBs were induced compared to the induction by CYN alone, suggesting antagonistic action of BPA against CYN in the mixture. The gene expression analysis, on the other hand, indicated additive or synergistic interactive effects in the CYN/BPs mixtures in several of the tested genes. The most pronounced effects were detected for the gene *CYP1A1*, where additive effects of CYN and BPs in all of the tested binary mixtures on gene up-regulation were indicated. In the case of the CYN/BPA mixture, the observed combined effect on *CYP1A1* up-regulation might have even been synergistic. The up-regulation of *CYP1A1* is of particular concern as the enzyme product of this gene catalyzes metabolic activation of pro-carcinogens to carcinogens that may result in increased susceptibility for genotoxic injury by indirect-acting genotoxic compounds to which humans can be exposed. The other genes, found differentially deregulated upon exposure to CYN/BPs mixtures compared to single compounds were *CDKN1A* and *GADD45A*, involved in DNA damage response, and *GCLC*, involved in oxidative stress response. The deregulation of these genes was more pronounced after exposure to the CYN/BPs binary mixtures compared to single compound exposure, suggesting an additive action of CYN and BPs. Our results confirm that co-exposure to pollutant mixtures can exert effects different from those caused by single compounds, even at relatively low concentrations, relevant for human exposure. Additionally, the results highlight the importance of co-exposure studies.

## 4. Materials and Methods

### 4.1. Chemicals

Cylindrospermopsin (CYN) was from Enzo Life Sciences GmbH (Lausen, Switzerland) (Table 1). A 0.5 mg/mL stock solution of CYN was prepared in 50% methanol and stored at −20 °C. Bisphenol A (BPA), bisphenol S (BPS), bisphenol F (BPF), and bisphenol AF (BPAF) were from Sigma-Aldrich (St. Louis, MO, USA) (Table 1). Stock solutions of all the BPs (25 mg/mL) were prepared in dimethylsulphoxide (DMSO): BPA (109.5 mM), BPS (99.89 mM), BPF (124.86 mM), and BPAF (74.35 mM), and stored at −20 °C. Minimal Essential Medium Eagle (MEM), non-essential amino acids (NEAA), Benzo(a)pyrene (BaP), methanol, DMSO, NaHCO3, phenazine methosulfate (PMS), and sodium pyruvate were from Sigma, USA. Penicillin/streptomycin, fetal bovine serum, L-glutamine, and phosphate-buffered saline (PBS) were from PAA Laboratories, USA. Etoposide (ET) was from Santa Cruz Biotechnology, USA. The CellTiter 96^®^ AQueous cell proliferation assay (3-(4,5-dimethylthiazol-2-yl)-2,5-diphenyltetrazolium bromide; MTS) was from Promega, Madison, WI, USA. Human recombinant Anti-H2AX pS139, FITC conjugate antibodies were from Miltenyi Biotec GmbH, Bergisch Gladbach, Germany. Trypsin was from Invitrogen™, Life Technologies, Waltham, MA, USA. The TRIzol^®^ reagent was from Thermo Fisher Scientific, Waltham, MA, USA. The cDNA High Capacity Archive Kit, TaqMan Universal PCR Master Mix, and Taq Man Gene Expression Assays (Table 1) were from Applied Biosystems, Waltham, MA, USA. All chemical reagents were of the purest grade available, and all solutions were made using Milli-Q water. The chemical structures shown in Table 1 were prepared with the ChemDraw Prime software from PerkinElmer Informatics, Waltham, MA, USA.

### 4.2. Cell Culture

The HepG2 cell line was obtained from the American Type Culture Collection (ATCC), USA, at passage 108. The cells were grown in MEM medium supplemented with 10% FBS, 2 mM L-glutamine, 1% NEAA, 2.2 g/L NaHCO3, 1 mM sodium pyruvate, and 100 IU/mL penicillin/streptomycin, at 37 °C and 5% CO_2_. Cell passages between 120 and 130 were used in the experiments. The cells were seeded on culture plates and left overnight to attach. Subsequently, the growth medium was replaced with fresh medium containing appropriate concentrations of single compounds CYN (0.5 µg/mL) and BPA, BPF, BPS, and BPAF (10 μg/mL) and binary mixtures of CNY and the different BPs, and incubated for 24 and 72 h. A negative control (growth medium containing 0.05% methanol and 0.04% DMSO) and assay-specific positive controls were included in all experiments. The final concentration of the solvent in the medium was adjusted in all experimental points.

### 4.3. Cell Viability—MTS Assay

Cell viability was studied after 24 and 72 h exposure of HepG2 cells to CYN, the BPs, and combinations thereof. ET (30 μg/mL) was used as a positive control. Cell viability was evaluated using the MTS tetrazolium reduction assay, as previously described by Hercog et al. [46]. Three independent experiments were performed, with five replicates per treatment point.

### 4.4. Analyses of the Induction of DNA DSB by the γ-H2AX Assay

Double-strand break (DSB) induction was evaluated through measuring γH2X formation, using flow cytometry (MACSQuant Analyzer 10; Miltenyi Biotech, Germany). The experiments were performed as described by Hercog et al. [46]. The cells were seeded on six-well plates (Corning Inc., USA), exposed to the tested compounds and their mixtures, and then collected and fixed using ethanol (70%). Fixed cells were washed and labeled with Anti-H2AX pS139 antibodies (130-118-339) according to the manufacturer’s protocol. Etoposide (ET, 1 μg/mL) was used as a positive control. FITC intensity was recorded for 10^4^ single cells in each sample. Independent experiments were repeated three times. GraphPad Prism 8 was used to generate box and whiskers plots. Statistical significance between treated groups and the vehicle control was determined with a linear mixed-effects model with the statistical program R [98] and its packages reshape [99], and nlme [100].

### 4.5. Real-Time Quantitative PCR (qPCR) Analysis

The mRNA expression of selected genes was analyzed by quantitative real-time PCR (qPCR). HepG2 cells were seeded on T25 plates (600,000 cells/plate) exposed to CYN, BPs alone, and to combinations thereof for 24 h. Total RNA was isolated using the TRIzol reagent according to the manufacturer’s protocol with minor modifications described by Maisanaba et al. [101]. The RNA was transcribed to cDNA using 1 μg of total RNA and cDNA High Capacity Archive Kit, according to the manufacturer’s protocol. Relative quantification of the selected genes was performed using qPCR, where the TaqMan Universal PCR Master Mix and Taqman Gene Expression Assays were used (Table 2).

*GAPDH* (VIC-TAMRA, Cat. No.:4310884E, Applied Biosystems, USA) was used as a reference gene in all experiments. All experiments were performed on 384-well plates, with a single probe per well using the ViiA™ 7 Real-Time PCR System (Applied Biosystems™). The conditions for the PCR were 50 °C for 2 min, 95 °C for 10 min, and 40 cycles of 95 °C for 15 s and 60 °C for 1 min. The relative quantification of gene expression was done by comparison of the Ct values of treated and control groups considering the actual efficiency of each assay using the Quant Genious protocol [102]. BaP (30 μM) was used as a positive control. Three independent experiments were performed each time in duplicates. Statistical difference between treated groups and controls was determined by two-tailed Student’s *t*-test. A ≥ 1.5-fold change in gene expression compared to control was considered as up-regulation and down-regulation, respectively.

## Figures and Tables

**Table 1:** CYN, BPA, and its structural analogues.

	Abbreviation	CAS N°	Chemical Structure	Formula	MW (g/mol)
**Cylindrospermopsin**	CYN	143545-90-8	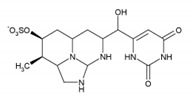	C_15_H_21_N_5_O_7_S	415.4
**Bisphenol A**	BPA	080-05-7	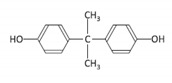	C_15_H_16_O_2_	228.29
**Bisphenol S**	BPS	080-09-1	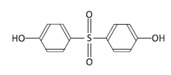	C_12_H_10_O_4_S	250.27
**Bisphenol F**	BPF	620-92-8	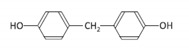	C_13_H_12_O_2_	200.23
**Bisphenol AF**	BPAF	1478-61-1	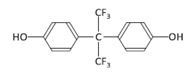	C_15_H_10_F_6_O_2_	336.23

**Table 2:** List of Taqman Gene Expression Assays used.

Gene Symbol	Assay ID	Entrez Gene Name	Cellular Function
*CYP1A1*	Hs01054797_g1	Cytochrome P450, family 1, subfamily A, polypeptide 1	Metabolism of xenobiotics, detoxification response
*GCLC*	Hs00155249_m1	Glutamate-cysteine ligase, catalytic subunit	Oxidative stress response
*GPX1*	Hs00829989_gH	Glutathione peroxidase 1	Oxidative stress response
*GSR*	Hs00167317_m1	Glutathione reductase	Oxidative stress response
*SOD1A*	Hs00533490_m1	Superoxide dis-mutase 1	Oxidative stress response
*CAT*	Hs00156308_m1	Catalase	Oxidative stress response
*CDKN1A*	Hs00355782_m1	Cyclin-dependent kinase inhibitor 1A (p21. Cip1)	DNA-damage response genes
*GADD45A*	Hs00169255_m1	Growth arrest and DNA damage-inducible, alpha	DNA-damage response genes
*MDM2*	Hs00234753_m1	MDM2 proto-oncogen	DNA-damage response genes
*TP53*	Hs01034249_m1	Tumor protein P53	DNA-damage response genes
*CHEK1*	Hs00967506_m1	Checkpoint kinase 1	DNA-damage response genes
